# What is the impact of n-3 PUFAs on inflammation markers in Type 2 diabetic mellitus populations?: a systematic review and meta-analysis of randomized controlled trials

**DOI:** 10.1186/s12944-016-0303-7

**Published:** 2016-08-20

**Authors:** Ning Lin, Jiao-jiao Shi, Yun-Ming Li, Xin-Yan Zhang, Yi Chen, Philip C. Calder, Li-Jun Tang

**Affiliations:** 1 Department of Clinical Nutrition, Chengdu Military General Hospital, Chengdu, 610083 China; 2 Department of Informatics, Chengdu Military General Hospital, Chengdu, 610083 China; 3 Faculty of Medicine, University of Southampton, Southampton, SO16 6YD UK; 4 NIHR Southampton Biomedical Research Centre, Southampton University Hospital NHS Foundation Trust and University of Southampton, Southampton, SO16 6YD UK; 5 Department of General Surgery, Chengdu Military General Hospital, Chengdu, 610083 China

**Keywords:** N-3 PUFAs, Inflammation markers, Type 2 diabetes mellitus, Fish oil

## Abstract

**Objectives:**

To explore the possible role of n-3 polyunsaturated fatty acids (PUFAs) in lowering inflammation markers in individuals with type 2 diabetes mellitus.

**Methods:**

PubMed, CNKI and Cochrane databases were searched until December 30, 2015; references from papers or reviews were also retrieved and screened. Screening was performed by two independent researchers, and randomized controlled trials reporting the specific n-3 PUFA type, dose, frequency, and duration of treatment, as well as the baseline and follow-up concentrations of inflammation markers, including interleukin 2 (IL-2), interleukin 6 (IL-6), tumor necrosis factor alpha (TNF-α) and C-reactive protein (CRP), were selected for final analysis. Data analysis was performed using RevMan 5.2 software.

**Results:**

Eight studies involving 955 participants were included; all reported CRP. Only one included study reported IL-2 or IL-6 while two studies reported TNF-α. N-3 PUFAs significantly reduced CRP concentration compared with control [SMD 95 % CI, 1.90 (0.64, 3.16), *Z* = 2.96, *P* = 0.003, random effect model].

**Conclusions:**

N-3 PUFAs decrease CRP concentration in type-2 diabetes mellitus. However, larger and rigorously designed RCTs are required to confirm this finding and extend it into other inflammatory biomarkers.

**Electronic supplementary material:**

The online version of this article (doi:10.1186/s12944-016-0303-7) contains supplementary material, which is available to authorized users.

## Background

Diabetes is a major global health concern with an increasing prevalence. It is estimated that by 2030, approximately 366 million adults will be diagnosed with diabetes worldwide [1]. There is a considerable health expenditure on the control of diabetes and the prevention of its comorbidities. Both type 1 and type 2 diabetes are associated with increased blood concentrations of several inflammatory biomarkers, including C-reactive protein (CRP), interleukin (IL)-2, IL-6 and tumour necrosis factor-alpha (TNF-α) [2–4]. Such low-grade inflammation, which may possibly be beneficial in the early stage for promoting β-cell proliferation and insulin production to compensate for insulin resistance, is the mechanism underlying insulin resistance [5].

Although they are strongly linked to chronic low-grade inflammation, diseases such as atherosclerosis, type 2 diabetes mellitus (T2DM) and obesity are not usually treated with anti-inflammatory pharmaceuticals. However, it is thought that many dietary factors can influence various aspects of inflammation either promoting or retarding specific inflammatory components [6]. Thus, nutrition may play a role in predisposing individuals to conditions that have an inflammatory component, and altered nutrition may be useful in preventing or treating such conditions.

Omega-3 (n-3) fatty acids are a family of polyunsaturated fatty acids (PUFAs) that are characterized by the position of a double bond in the hydrocarbon (acyl) chain being between carbon numbers 3 and 4 counting the terminal methyl carbon as number one. Longer chain n-3 fatty acids include eicosapentaenoic acid (EPA; 20:5n-3), docosapentaenoic acid (DPA; 22:5n-3) and docosahexaenoic acid (DHA; 22:6n-3) [7]. Epidemiological, human intervention, animal and cell culture studies have provided evidence supporting a beneficial role for dietary n-3 PUFAs in many conditions associated with low-grade chronic inflammation [6, 8–10]. N-3 PUFAs can potentially decrease inflammation through several mechanisms, including inhibition of the arachidonic acid (AA) pathway, inhibition of nuclear factor kappa B activation, and initiation of anti-inflammatory signalling through G-protein coupled receptor 120 [11–13].

Whether n-3 PUFAs favorably affect biomarkers of inflammation in people with T2DM is not clear. Our aim was to perform a systematic review and meta-analysis to investigate whether n-3 PUFAs affect inflammation markers in T2DM.

## Materials and methods

### Literature search

All English and Chinese language literature published before December 30, 2015, was retrieved from PubMed, China National Knowledge Infrastructure (CNKI), and Cochrane library databases, and RCTs involving n-3 PUFAs and patients with T2DM were independently screened for by two researchers. The following search strategy was used:

- # 1 ((“fatty acids”[MeSH Terms] OR (“fatty”[All Fields] AND “acids”[All Fields]) OR “fatty acids”[All Fields]))
- # 2 n3 [All Fields] AND #1
- # 3 (“fatty acids, omega-3” [MeSH Terms] OR (“fatty” [All Fields] AND “acids” [All Fields] AND “omega-3” [All Fields]) OR “omega-3 fatty acids” [All Fields] OR “omega 3 fatty acids” [All Fields])),
- # 4 (“fish oils” [MeSH Terms] OR (“fish” [All Fields] AND “oils” [All Fields]) OR “fish oils” [All Fields] OR (“fish” [All Fields] AND “oil” [All Fields]) OR “fish oil” [All Fields]),
- # 5 “#2” OR “# 3” OR “# 4”,
- # 6 (“inflammation” [MeSH Terms] OR “inflammation” [All Fields]),
- # 7 Clinical Trial [ptyp],
- # 8 “#5” AND “# 6” AND “# 7”.

Titles and abstracts were obtained for selected articles. Citation indices and reference lists of retrieved articles were checked for additional studies that were not identified in the original database search.

### Study selection

In phase 1, all papers retrieved by an independent author were reviewed by at least two other authors who independently selected the papers that they believed met the inclusion criteria for full-text reading. In cases of disagreement, the researchers discussed their different assessments and reached a consensus. In phase 2, the first author reviewed all full articles that met the eligibility criteria. The following inclusion criteria were applied:

- a study should report the baseline and after-treatment data for at least one inflammation biomarker;
- all study subjects should be diagnosed or confirmed with T2DM, with reported impaired fasting glucose;
- a control arm, with or without a placebo, should be described.

After confirming the included literature, baseline and post-treatment inflammation biomarker data were selected and placed in a PICOS table that included the author names, publication year, participant characteristics, intervention and controlled arms, and n-3 PUFA dose, as well as the control, baseline and end-point levels of each reported inflammation biomarker.

### Data collection and calculation

If the required data could not be directly gathered from the paper, the corresponding author was contacted by email to request these details. The Δ change value of any inflammation marker equals the after-treatment value minus the baseline value. Finally, the Δ change of the inflammation markers in the n-3 PUFA and control arms were compared. If the Δ change value was reported in the paper or as supplementary material, the data were directly used for the analysis.

If a study reported inflammation markers at two or more time points, for example at 12 and 16 weeks of intervention, we regarded them as two subgroups.

### Quality evaluation

Clinical trial bias and the quality of the literature were evaluated by two independent researchers, according to the Cochrane risk of bias guidelines [14], which included random sequence generation, allocation concealment, blinding of participants, personnel and outcome assessors, incomplete outcome data, selective outcome reporting, and other bias measurements (Additional file 2).

### Statistical analysis

For continuous variables, the pooled effect was reported as the standardized mean difference (SMD), with the corresponding 95 % CI [15]. Heterogeneity was assessed using I2 tests, and a *p* value of < 0.05 was considered to be significant. If heterogeneity was present, the pooled effect size was calculated through a random-effects model.

## Results

### Search results and study characteristics

**Fig. 1:**
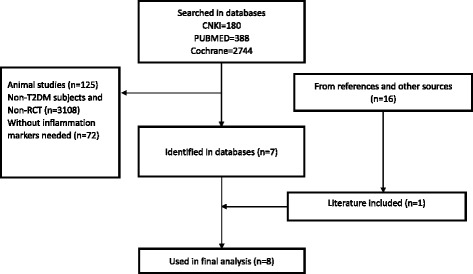
Flow chart for selection of included studies

We identified 3312 published papers from three databases (Fig. 1). Review articles, viewpoints, editorials, commentaries, and animal studies were excluded. Clinical trials that did not provide data on the effect of n-3 PUFAs on any of the evaluated inflammation markers were also excluded. According to the inclusion and exclusion criteria, 8 RCTs [16–23] were selected for inclusion (Fig. 1, Additional files 1 and 2). Reference papers identified in other reviews of n-3 PUFAs and T2DM were also retrieved, but none were included in the final analysis. In total, data from 8 RCTs in T2DM were included in the analysis.

### Effect of N-3 PUFAs on inflammation markers

Eight RCTs were selected for the final analysis; these included 955 participants (Table 1). In all eight studies the age of all participants was over 18 y. The duration of n-3 PUFA intervention ranged from 6 to 12 weeks. Either fish oil providing a mix of EPA and DHA or pure EPA or DHA were used and in all studies these were provided in capsules. The minimum daily dose of n-3 PUFAs used was 1 g and the maximum was 6 g (Table 1). Data for inflammation biomarkers from the eight included studies are shown in Table 2.

**Table 1:** Characteristics of included studies regarding n-3 PUFA from any source and inflammation markers in T2DM

							Intervention arm		Control arm	
Author/year [reference number]	Study type	Type of patient	Location of study	Number included/Number completed	Age (y) (Mean ± SD)	Duration (wk)	N-3 PUFA source	Dose (g/d)	Placebo	Quality
Brinton 2013 [21]	RCT	T2DM	USA	513/501	>18	12	Icosapent ethyl (EPA)	4, 2	placebo	A
Azizi-Soleiman 2013 [16]	RCT	T2DM	Iran	60/45	59.4 ± 8.2	12	EPA or DHA	1	Canola oil	B
Lee 2014 [17]	RCT	Early-stage T2DM or MetS	USA	80/59	57.9	8	EPA + DHA	6	Corn oil	B
Malekshahi Moghadam 2012 [18]	RCT	T2DM	Iran	84/NA	45–85 (mean 54.2)	8	EPA + DHA	2.7	Sunflower oil	B
Mori 2003 [19]	RCT	Treated-hypertensive T2DM	Australia	59/51	61.2 ± 1.2	6	EPA or DHA	4	Olive oil	C
Pooya 2008 [20]	RCT	T2DM	Iran	90/81	45–85 (mean 54.5)	8	EPA + DHA	2.2	Sunflower oil	B
Soleimani 2015 [23]	RCT	T2DM with diabetic nephropathy (DN)	Iran	60/60	45–85 (mean 62.6)	12	Flaxseed oil (ALA)	1	placebo	B
Wong 2015 [22]	RCT	T2DM without prior cardiovasular disease	China	97/91	60 ± 9 (Mean 60.1)	12	Fish oil (42 % EPA +25 % DHA)	4	Olive oil	A

*MetS* metabolic syndrome, *RCT* randomized controlled trial, *T2DM* type 2 diabetes mellitus, *NA* not given, *ALA* alpha-linolenic acid

**Table 2:** Inflammation biomarkers pre- and post-intervention across included studies

		Placebo			N-3 PUFA				
Inflammation biomarker	Author/year	n	Pre-intervention	Post-intervention	n	Pre-intervention	Post-intervention	P (vs placebo)	Note
CRP (mg/l)	Brinton 2013 [21]	165	2.50 ± 2.88	3.10 ± 3.48	165	2.50 ± 2.07	2.10 ± 2.52	NA	4 g/d EPA
		165	2.50 ± 2.88	3.10 ± 3.48	171	2.10 ± 2.67	2.70 ± 2.52	NA	2 g/d EPA
	Azizi-Soleiman 2013 [16]	17	2.15 ± 2.29	2.42 ± 2.49	14	2.20 ± 2.65	2.73 ± 3.45	NA	EPA treatment
		17	2.15 ± 2.29	2.42 ± 2.49	14	2.85 ± 3.58	2.18 ± 2.98	NA	DHA treatment
	Lee 2014 [17]	21	2.36 ± 0.57	3.72 ± 1.00	16	6.08 ± 3.19	3.59 ± 1.03	NA	
	Malekshahi Moghadam 2012 [18]	42	18.7 ± 16.8	18.2 ± 11.1	42	25.7 ± 27.4	20.4 ± 24.2	>0.05	
	Mori 2003 [19]	16	2.01 ± 0.57	2.12 ± 0.57	17	2.28 ± 0.55	2.02 ± 0.43	>0.05	EPA treatment
		16	2.01 ± 0.57	2.12 ± 0.57	17	3.70 ± 0.61	3.84 ± 0.73	>0.05	DHA treatment
	Pooya 2008 [20]	41	3.15 ± 0.36	3.80 ± 0.17	40	2.70 ± 0.02	2.48 ± 0.23	>0.05	
	Soleimani 2015 [23]	30	2.93 ± 0.45	2.56 ± 0.43	30	2.66 ± 0.51	2.42 ± 0.45	NA	
	Wong 2015 [22]	48	1.68 ± 2.53	1.28 ± 1.56	49	1.36 ± 1.43	1.71 ± 3.11	0.15	No adjustment for baseline value
TNF-α (pg/ml)	Malekshahi Moghadam 2012 [18]	42	38.7 ± 9.53	40.6 ± 11.0	42	37.5 ± 6.41	34.5 ± 6.40	0.002	
	Mori 2003 [19]	16	15.35 (10.7–22.0)	14.26 (10.7–18.9)	17	24.44 (18.2–32.9)	19.67 (14.6–26.5)	>0.05	EPA treatment
		16	15.35 (10.7–22.0)	14.26 (10.7–18.9)	17	20.51 (14.7–28.6)	13.78 (9.50–19.9)	>0.05	DHA treatment
IL-2 (pg/ml)	Malekshahi Moghadam 2012 [18]	42	42.46 ± 19.78	51.52 ± 19.71	42	42.47 ± 13.85	35.25 ± 11.28	0.0001	
IL-6 (pg/ml)	Mori 2003 [19]	16	1.76 (1.39–2.23)	1.96 (1.57–2.45)	17	1.75 (1.40–2.20)	1.78 (1.54–2.06)	>0.05	EPA treatment
		16	1.76 (1.39–2.23)	1.96 (1.57–2.45)	17	2.22 (1.46–3.38)	2.15 (1.52–3.03)	>0.05	DHA treatment

Mean ± SD or Geometric mean (95 % confidence interval); For Azizi-Soleiman (2013) we adjusted the reported units for CRP concentration; *NA* not available

**Fig. 2:**
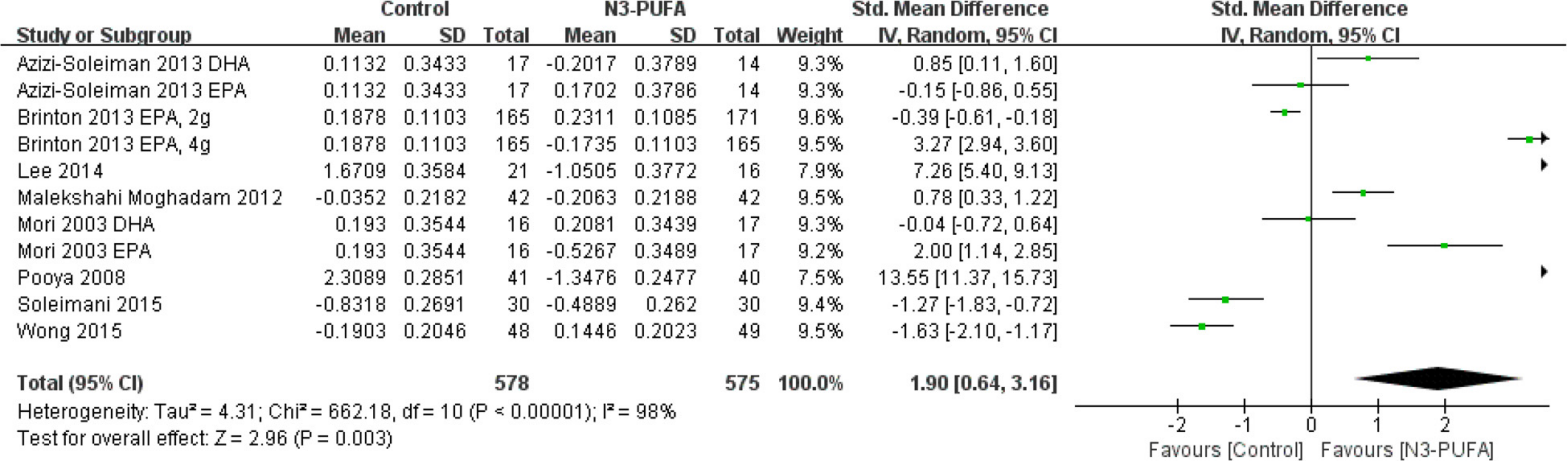
Forest plot for effect of n-3 PUFA on CRP concentration in all studies (random effect model)

A pooled analysis of the 8 included studies [16–23] enrolling 955 T2DM subjects revealed that the concentration of CRP was significantly decreased in the n-3 PUFA group compared with the control group (SMD 1.90; 95 % CI, 0.64 to 3.16, *p* = 0.003; Fig. 2. The test for heterogeneity was significant (*I*^2^ = 98 %, *p* < 0.00001).

**Fig. 3:**
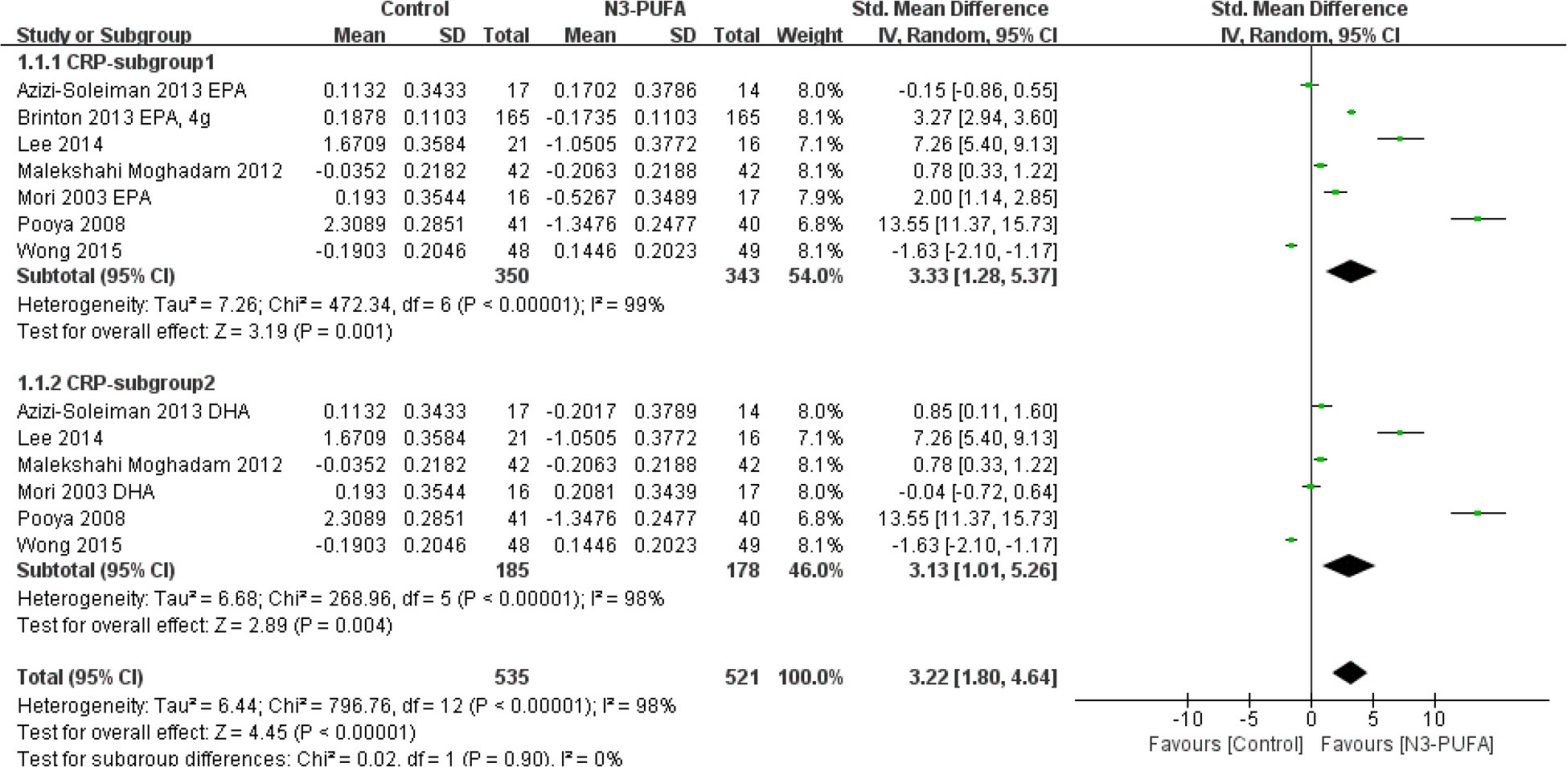
Subgroup analysis of n-3 PUFA on CRP

For a subgroup analysis, EPA and DHA were tested separately (Fig. 3). Although the heterogeneity was significant in both subgroups, the *P* values for the SMD in each analysis were less than 0.05 indicating that both EPA and DHA lower CRP concentration.

Only two trials reported data for TNF-α concentration. Malekshahi Moghadam [17] found that n-3 PUFA intake at 2.7 g/d significantly lowered TNF-α concentration compared to sunflower oil as placebo. However, Mori [19] reported that neither EPA nor DHA at 4 g/d lowered TNF-α concentration in early-stage T2DM patients.

Only one trial explored the effects of n-3 PUFAs on IL-2 and IL-6 in T2DM [18, 19]. IL-2 concentration was significantly decreased in T2DM subjects who underwent an 8-week intervention with 2.7 g/d n-3 PUFAs. No significant effect of n-3 PUFAs on IL-6 concentration was detected.

## Discussion

The overall findings from this systematic review and meta-analysis suggest that supplemental n-3 PUFAs can lower CRP concentration in patients with T2DM, although the heterogeneity was significant among the selected studies. There were too few studies to evaluate the effect of n-3 PUFAs on other inflammatory biomarkers in T2DM to reach any clear conclusion.

CRP, along with IL-2, IL-6 and TNF-α, belongs to the cluster of general inflammation markers identified in a large variety of diseases and conditions. Clinically, CRP has emerged as a leading inflammatory biomarker for CVD [24] and, thus, has been of interest to researchers studying the role of n-3 PUFAs in diabetes complications, a key CVD risk factor. Epidemiological studies have assessed the relationship between CRP concentration and n-3 fatty acid intakes in healthy subjects and in patients with coronary disease [24, 25], confirming an inverse association and suggesting an anti-inflammatory effect of n-3 PUFAs. EPA and DHA can both decrease TNF-α levels and improve insulin resistance [26, 27]. In Mori’s study, both EPA and DHA tended to decrease the inflammatory marker TNF-α, although this did not reach statistical significance [19]. In vitro and in vivo studies of n-3 and n-6 fatty acids have revealed that these fatty acid families could inhibit the production of inflammatory cytokines, including IL-1, IL-2 and TNF-α, by stimulated human lymphocytes [28]. EPA and DHA were found to markedly lower IL-2 levels when incubated with lymphocytes from diabetic patients or from controls [29].

Although several studies support the idea that n-3 PUFAs can potentially lower inflammation markers, the findings are inconsistent. There are several possible reasons for this. First, the type of subjects studied varies from trial to trial and n-3 PUFAs might be less effective in some types of subject. For instance, one study found that consumption of n-3 PUFAs did not affect the plasma levels of CRP among individuals who suffer from dyslipidaemia and obesity [30]. In contrast, another study reported that 4 weeks of n-3 PUFA supplementation lowered serum CRP levels in individuals with inflammatory diseases by 93 % [31]. Secondly, dose-dependent actions of n-3 PUFAs on inflammatory responses have not been well described, but it appears that a dose of at least 2 g/d is necessary to achieve an anti-inflammatory effect [32]. Studies included in the current meta-analysis used a variety of doses, some below the suggested anti-inflammatory threshold. Thirdly, EPA and DHA may have different effects and different studies have used different mixtures of those two fatty acids. Azizi-Soleiman et al. showed that 1 g/d of DHA but not EPA was able to lower CRP concentration [16]. Fourthly, small patient cohorts, short study durations and genetic variability may also play roles in the inconsistencies. An individual’s genetic background may affect the response to fish oil supplementation. In Grimble’s study, SNPs at -308 and +252, respectively, of the TNF-α and LT-α genes were found to influence the ability of fish oil (a source of n-3 PUFAs) to suppress TNF-α production in a complex manner [33].

In addition, circulating levels of CRP and IL-6 and also of adiponectin are beneficially modulated by weight loss and (or) exercise [34–36]. Studies of n-3 PUFA supplementation tend not to consider body weight, BMI or/and exercise or adjust for these factors as covariates [37].

This current study has some limitations. First, the number of included studies is low, which could give rise to publication bias. Secondly, literature published only in English or Chinese was considered, which may be a selection bias.

## Conclusions

In conclusion, this meta-analysis indicates that persons with T2DM who received n-3 PUFA supplements had significantly lower CRP levels compared with subjects in control groups. However, the number and scale of the included studies were small. Further carefully designed RCTs are required to confirm this finding and extend it into other inflammatory biomarkers.

## Additional files

Additional file 1: PRISMA 2009 Checklist detail. (DOC 65 kb) Additional file 2: Quality and study bias of selected literature. (DOCX 14 kb)
