# Supplementary material for: What is the impact of n-3 PUFAs on inflammation markers in Type 2 diabetic mellitus populations?: a systematic review and meta-analysis of randomized controlled trials
Source: Lipids Health Dis. 2016 Aug 20;15:133. doi: 10.1186/s12944-016-0303-7 (PMC4992564; doi:10.1186/s12944-016-0303-7)
Supplement: Additional file 2: — Quality and study bias of selected literature. (DOCX 14 kb) [file 12944_2016_303_MOESM2_ESM.docx]

Quality and study bias of selected literature

| study | Random sequence generation | Allocation concealment | Blinding of participants and personnel | Blinding of outcome assessment | Incomplete outcome data | Selective reporting |
| --- | --- | --- | --- | --- | --- | --- |
| Brinton 2013 | Low risk | Low risk | Low risk | Low risk | Low risk | high risk |
| Fatemeh 2013 | Low risk | High risk | High risk | Low risk | high risk | Low risk |
| Lee 2014 | Low risk | High risk | High risk | High risk | Low risk | Low risk |
| Moghadam 2012 | Low risk | High risk | Low risk | Low risk | high risk | Low risk |
| Mori 2003 | Low risk | High risk | High risk | Low risk | high risk | Low risk |
| Pooya 2008 | Low risk | High risk | Low risk | Low risk | high risk | Low risk |
| Soleimani 2015 | Low risk | Low risk | Low risk | Low risk | Low risk | Low risk |
| Wong 2015 | Low risk | low risk | Low risk | Low risk | low risk | high risk |
